# Supplementary material for: Guanylate binding proteins directly attack Toxoplasma gondii via supramolecular complexes
Source: eLife. 2016 Jan 27;5:e11479. doi: 10.7554/eLife.11479 (PMC4786432; doi:10.7554/eLife.11479)
Supplement: Supplementary file 1. — The residues used to define the dipole of the chromophoric groups are indicated. (b) Calculations of donor-acceptor distances (Rsim) and orientation factors (κ2) from each sampled conformation from MC molecular simulation of G-mGBP2/mCh-mGBP2 dimer in steps. See Experimental procedures and Figure 7—figure supplement 1 for details. DOI: http://dx.doi.org/10.7554/eLife.11479.027 [file elife-11479-supp1.docx]

**Supplementary file 1a.** Amino-acid sequence settings in the MC molecular simulation. The residues used to define the dipole of the chromophoric groups are indicated.

| GFP | kept rigid | MVSKGEELFTGVVPILVELDGDVNGHKFSVSGEGEGDATYGKLTLKFICTTGKLPVPWPTLVTTLTYGVQCFSRYPDHMKQHDFFKSAMPEGYVQERTIFFKDDGNYKTRAEVKFEGDTLV**N**RIELKGIDFKEDGNILGHKLEYNY**N**SHNVYIMADKQKNGIKVNFKIRHNIEDGSVQLADHYQQNTPIGDGPVLLPDNHYLSTQSALSKDPNEKRDHMVLLEFVTAA |
| --- | --- | --- |
|  | flexible | GITLGMDELYKSGLRSELNFEFPGASEIHMSEP |
| mGBP2 | kept rigid | MCLIENTEAQLVINQEALRILSAITQPVVVVAIVGLYRTGKSYLMNKLAGKRTGFSLGSTVQSHTKGIWMWCVPHPKKAGQTLVLLDTEGLEDVEKGDNQNDCWIFALAVLLSSTFIYNSIGTINQQAMDQLHYVTELTDLIKSKSSPDQSGVDDSANFVGFFPTFVWTLRDFSLELEVNGKPVTSDEYLEHSLTLKKGADKKTKSFNEPRLCIRKFFPKRKCFIFDRPAQRKQLSKLETLREEELCGEFVEQVAEFTSYILSYSSVKTLCGGIIVNGPRLKSLVQTYVGAISNGSLPCMESAVLTLAQIENSAAVQKAITHYEEQMNQKIQMPTETLQELLDLHRPIESEAIEVFLKNSFKDVDQKFQTELGNLLVAKRDAFIKKNMDVSSARCSDLLEDIFGPLEEEVKLGTFSKPGGYYLFLQMRQELEKKYNQAPGKGLQAEAMLKNYFDSKADVVETLLQTDQSLTEAAKEVEEERTKAEAAEAANRELEKKQKEFELMMQQKEKSYQEHVKKLTEKMKDEQKQLLAEQENIIAAKLREQEKFLKEGFENESKKLIREIDTLKQNKSSGKCTIL |

| mCherry | kept rigid | MVSKGEEDNMAIIKEFMRFKVHMEGSVNGHEFEIEGEGEGRPYEGTQTAKLKVTKGGPLPFAWDILSPQFMYGSKAYVKHPADIPDYLKLSFPEGFKWERVMNFEDGGVVTVTQDSSLQDGEFI**Y**KVKLRGTNFPSDGPVMQKKTMGW**E**ASSERMYPEDGALKGEIKQRLKLKDGGHYDAEVKTTYKAKKPVQLPGAYNVNIKLDITSHNEDYTIV |
| --- | --- | --- |
|  | flexible | EQYERAEGRHSTGGMDELYKEFPGASEIHMSEP |
| mGBP2 | kept rigid | MCLIENTEAQLVINQEALRILSAITQPVVVVAIVGLYRTGKSYLMNKLAGKRTGFSLGSTVQSHTKGIWMWCVPHPKKAGQTLVLLDTEGLEDVEKGDNQNDCWIFALAVLLSSTFIYNSIGTINQQAMDQLHYVTELTDLIKSKSSPDQSGVDDSANFVGFFPTFVWTLRDFSLELEVNGKPVTSDEYLEHSLTLKKGADKKTKSFNEPRLCIRKFFPKRKCFIFDRPAQRKQLSKLETLREEELCGEFVEQVAEFTSYILSYSSVKTLCGGIIVNGPRLKSLVQTYVGAISNGSLPCMESAVLTLAQIENSAAVQKAITHYEEQMNQKIQMPTETLQELLDLHRPIESEAIEVFLKNSFKDVDQKFQTELGNLLVAKRDAFIKKNMDVSSARCSDLLEDIFGPLEEEVKLGTFSKPGGYYLFLQMRQELEKKYNQAPGKGLQAEAMLKNYFDSKADVVETLLQTDQSLTEAAKEVEEERTKAEAAEAANRELEKKQKEFELMMQQKEKSYQEHVKKLTEKMKDEQKQLLAEQENIIAAKLREQEKFLKEGFENESKKLIREIDTLKQNKSSGKCTIL |

**Supplementary file 1b.** Calculations of donor-acceptor distances (*R_sim_*) and orientation factors (κ^2^) from each sampled conformation from MC molecular simulation of G-mGBP2/mCh-mGBP2 dimer in steps. See Experimental procedures and Figure S7 for details.

|  | Donor (GFP) | Acceptor (mCherry) |
| --- | --- | --- |
| Coordinates of the two chosen C_α_-atoms | $\vec{r}_{D1}$ and $\vec{r}_{D2}$ | $\vec{r}_{A1}$ and $\vec{r}_{A2}$ |
| Distance between the two C_α_-atoms |  |  |
| Unit vector connecting the two C_α_-atoms |  |  |
| Coordinates of the middle point of the connecting vector |  |  |
| Calculation of donor-acceptor distance and orientation factor |    | |
